# ﻿*Coptisaustrogaoligongensis* (Ranunculaceae), a new species from West Yunnan, China

**DOI:** 10.3897/phytokeys.244.127978

**Published:** 2024-07-19

**Authors:** Zhuo Cheng, Jiahua Li, Congli Xu, Lixiang Zhang, Qiangbang Gong, Chunlin Long

**Affiliations:** 1 Key Laboratory of Ecology and Environment in Minority Areas (Minzu University of China), National Ethnic Affairs Commission of China, Beijing, 100081, China Minzu University of China Beijing China; 2 College of Life and Environmental Sciences, Minzu University of China, Beijing, 100081, China Gaoligongshan National Nature Reserve Yunnan China; 3 Gaoligongshan National Nature Reserve (Longyang Sub-bureau), Yunnan, 678000, China Yunnan Tongbiguan Provincial Nature Reserve Management and Protection Bureau Yunnan China; 4 Gaoligongshan National Nature Reserve (Baoshan Bureau), Yunnan, 678000, China Minzu University of China Beijing China; 5 Yunnan Tongbiguan Provincial Nature Reserve Management and Protection Bureau, Yunnan, 678400, China Gaoligongshan National Nature Reserve Yunnan China; 6 Institute of National Security Studies, Minzu University of China, Beijing, 100081, China Yunnan Tongbiguan Provincial Nature Reserve Management and Protection Bureau Yunnan China

**Keywords:** *Coptis*, *Coptisaustrogaoligongensis*, taxonomy, Yunnan Province

## Abstract

Based on morphological and plastid data, we have described and confirmed that *Coptisaustrogaoligongensis* distributed in Tongbiguan Provincial Nature Reserve, Yingjiang County, Yunnan Province, is a new species of *Coptis*. It is distinctly different from C.teetasubsp.teeta and C.teetasubsp.lohitensis with differences mainly reflected in the following features: former leaf segment lobes contiguous to each other, and lateral segments equal to central one; plants without developed stolons; inflorescences with only 1–3 flowers; petals have short claws. Phylogenetic analysis indicated that *C.austrogaoligongensis* is a sister to C.teetasubsp.teeta and C.teetasubsp.lohitensis.

## ﻿Introduction

*Coptis* Salisb. is a small genus of Ranunculaceae, consisting of perennial herbs with yellowish rhizomes and numerous fibrous roots (Wang 1979, 2001). The taxonomy of *Coptis* has traditionally been based on vegetative (leaf type and shape) and reproductive (flower number, color and shape of sepals and petals, and beak length) characters (Xiang et al. 2016, 2018). The genus *Coptis* comprises 17 species worldwide, which is mainly distributed in the warm temperate to the cold coniferous forests of eastern Asia and North America (Xiang et al. 2016, 2018; Wang et al. 2020). Representatives of the genus *Coptis* are considered essential medicinal plants in Eastern Asia. They are rich in isoquinoline type alkaloids, such as berberine, epiberberine, coptisine, palmatine, jatrorrhizine, and columbamine, and their dried rhizomes are used in traditional Chinese medicine as *Rhizoma Coptidis*, which is famous for clearing heat, removing dampness, and reducing fire (Wang et al. 2019; Yang et al. 2021).

According to the morphology, especially flower and leaf, genus representatives distributed in China had been classified into seven species, one variant and one subspecies, i. e., *C.chinensis* Franch. (endemic to SW China), C.chinensisvar.brevisepala W. T. Wang & P. K. Hsiao (endemic to SE China), *C.deltoidea* C. Y. Cheng & P. K. Hsiao (endemic to Sichuan, China), *C.omeiensis* (Chen) C. Y. Cheng (endemic to Sichuan, China), *C.quinquefolia* Miq. (distributed in Taiwan Province and Japan), *C.quinquesecta* W. T. Wang (endemic to Yunnan, China), *C.teeta* Wall. (distributed in East Himalaya), C.teetasubsp.lohitensis Pandit & Babu (distributed in East Himalaya), and *C.huanjiangensis* L.Q. Huang, Q.J. Yuan & Y.H. Wang. *C.huanjiangensis* as a new species was described during the survey of traditional Chinese medicine resources in Huanjiang, Guangxi in 2022 (Wang et al. 2022).

*Coptisteeta* complex is an important local medicinal plant in China. The *C.teeta* complex is divided into two subspecies, C.teetasubsp.teeta and C.teetasubsp.lohitensis, based on the presence of stolons and reproduction methods (Pandit and Babu 1993, 2000, 2003). Coptisteetasubsp.teeta is mainly distributed in Changdu and Linzhi of Xizang, China. It used as a medicinal plant by the Monba and Lhoba ethnic groups in southeastern Xizang. C.teetasubsp.lohitensis is mainly distributed in the Gaoligong Mountains in the northwest of Yunnan Province. It was used as a currency equivalent exchange in the past. C.teetasubsp.lohitensis is used in the treatment of diarrhea, dysentery and eye diseases by the Lisu, Dulong and Nu ethnic minorities in Gaoligong Mountain of Yunnan Province (Cheng et al. 2022, 2024).

In March 2023, during the investigation of *C.teeta* resources in southeast Tibet and northwest Yunnan, we found one population in Yingjiang County of Dehong Dai and Jinpo Autonomous Prefecture, which was different from the previously observed materials of *C.teeta* complex. The difference was mainly reflected in the fact that leaf segment lobes were contiguous to each other, and lateral segments equal to central one. Plants had no developed stolons. But there was no option to evaluate the morphological features of flowers at that time. In March 2024, we obtained flowers material. It was found that inflorescence consists of 1–3 flowers. The petals have short claws. This was obviously different from C.teetasubsp.teeta and C.teetasubsp.lohitensis characteristics. At the same time, we also collected materials for the molecular studies, and the results of consequent phylogenetic analysis proved that this population is the sister group of C.teetasubsp.teeta and C.teetasubsp.lohitensis.

## ﻿Methods

### ﻿Material sampling and DNA extraction

Samples of the new species were collected from Yingjiang County, Dehong Dai and Jinpo Autonomous Prefecture. The plastome sequences of 8 related *Coptis* species (a total of sixteen accessions) and an outgroup species were obtained from GenBank (http://www.ncbi.nlm.nih.gov). The total genomic DNA was extracted from the fresh leaves using the modified CTAB method (Doyle and Doyle 1987), and libraries were prepared using the TruePrep DNA Library Prep Kit (Vazyme Biotech Co., Ltd, Nanjing, CN). All the DNA and molecular materials were deposited in the herbarium of Minzu University of China (MUC). Sample information is listed in Suppl. material 1: table S1. For principal component analysis (PCA), we measured more than 20 individuals with complete traits. In our examination, we focused on 19 morphological characters, which also encompassed both vegetative and reproductive characteristics (Suppl. material 1: table S2). These characters were chosen based on their relevance in species identification and establishment, as described by Sun and Zhang (1995).

### ﻿Plastome sequencing and assembly

Genomic paired-end sequencing was conducted using the Illumina Novaseq 6000 platform. The chloroplast genome was assembled and analyzed using the program NOVOPlasty v. 4.3.1 (Dierckxsens et al. 2017). Annotation was performed with CPGView to determine the initial location of the chloroplast genome and the IR region (Liu et al. 2023), with the chloroplast genome of *C.teeta* (NC 054331) serving as a reference. The annotations were manually checked for errors using Zhou et al. (2021) as reference. The final chloroplast genome of new species was deposited in the NCBI GenBank under accession numbers: PP786562 and PP786563.

### ﻿Phylogenetic reconstruction

Fifty-six single copy protein-coding genes (PCGs) were extracted from 19 chloroplast sequences using the PhyloSuite v. 1.2.3 software (Zhang et al. 2020; Xiang et al. 2023). They were aligned using the MAFFT v. 7.149b algorithm (Katoh et al. 2019). All these single gene alignments were concatenated to create a document for phylogenetic analyses. The best-fit model was determined using the Akaike information criterion (AIC) in ModelFinder program (Kalyaanamoorthy et al. 2017). To determine its phylogenetic position, a maximum likelihood (ML) tree was constructed by IQ-TREE v. 1.6.10. Bayesian inference (BI) analysis was performed with MrBayes based on 56 PCGs of 8 other *Coptis* species through PhyloSuite v. 1.2.3 software. Phylogenetic trees were visualized, rooted with *Asteropyrumpeltatum*, and edited using the iTOL v. 5 (Ivica and Peer 2021). R v.4.3.2 was employed for data analysis, the ggplot2 package was used for statistical chart visualization, and the factoextra package was used for presenting the PCA plot charts.

## ﻿Results

### ﻿Phylogenetic and morphological analysis

**Figure 1.**
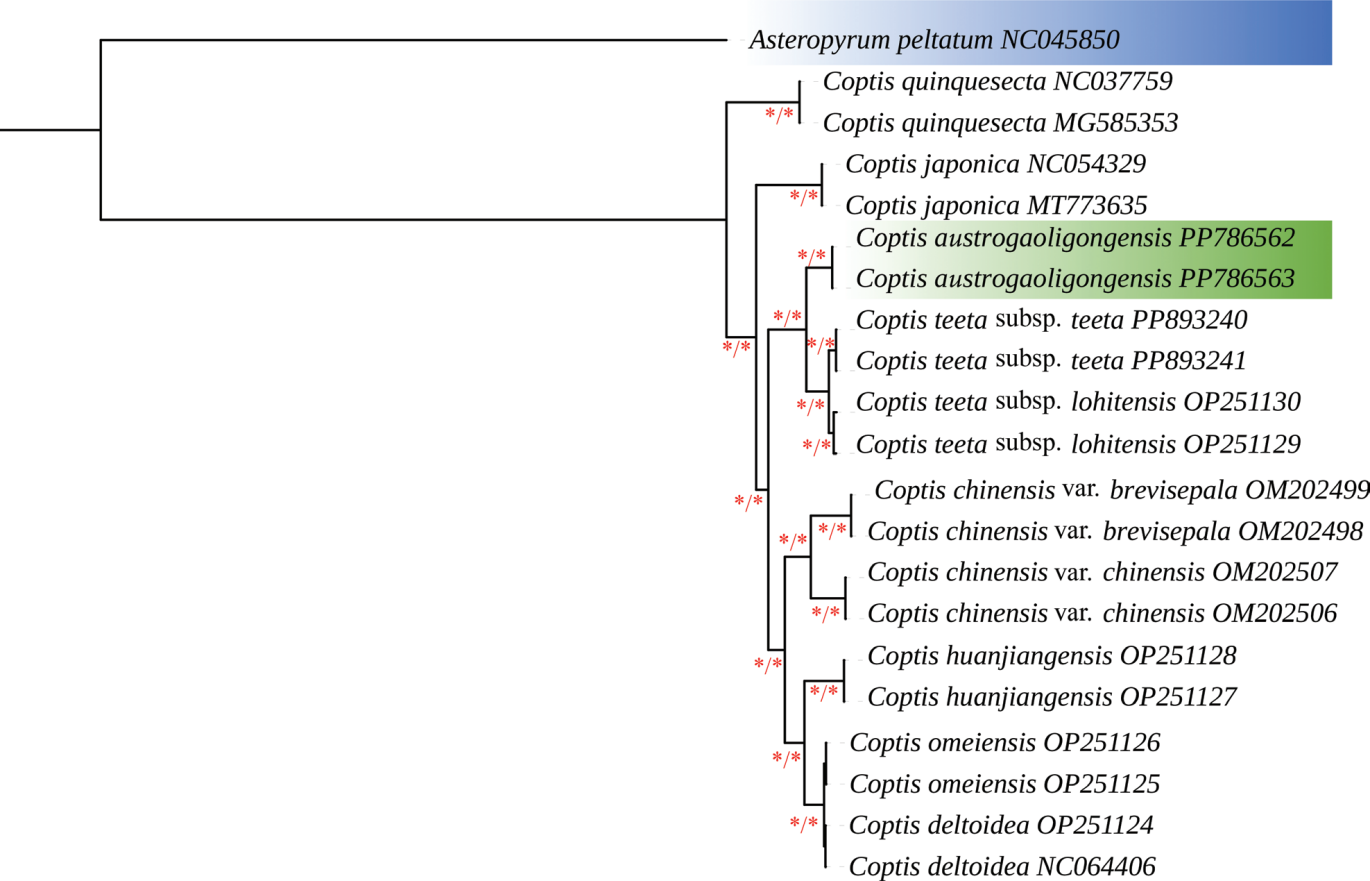
Consensus phylogenetic tree reconstructed by ML and BI analysis based on 56 protein-coding sequences (CDS) of 10 species, with *Asteropyrumpeltatum* as outgroups. Asterisks near the branches indicate bootstrap support (BS) percentages obtained from maximum likelihood inference and posterior probabilities (PP) obtained from Bayesian analysis (BS/PP). Those nodes with BS≥90%, PP =1.00 were shown with asterisks.

**Figure 2.**
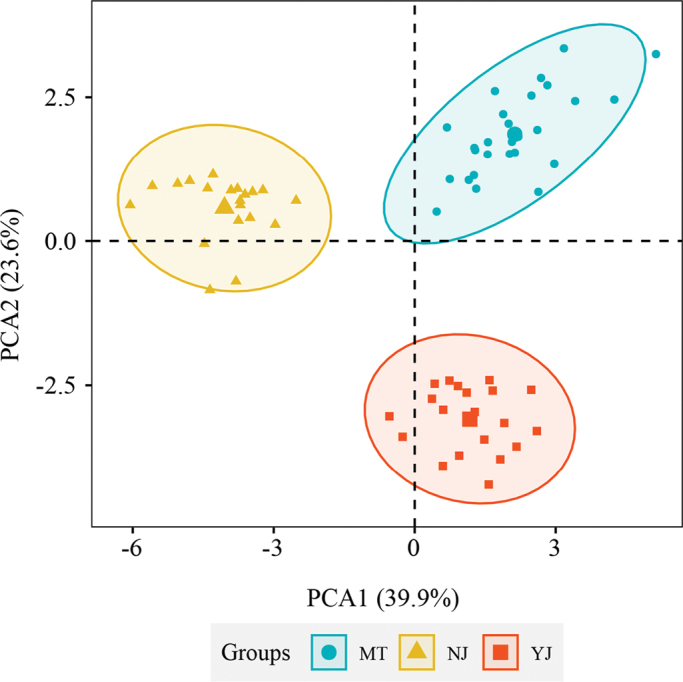
Morphological principal component analysis (PCA) of three species based on some individuals (20 NJ individuals, 20 YJ individuals, and 27 MT individuals) and 19 morphological traits. MT: C.teetasubsp.teeta, NJ: C.teetasubsp.lohitensis, YJ: *C.austrogaoligongensis*.

Consensus phylogenetic tree reconstructed by ML and BI analyze based on 56 PCGs of 10 species, with *Asteropyrumpeltatum* as outgroups is represented in the Fig. 1. The topologies of the ML and BI trees were identical with all the branches strongly-supported (ML BS≥90% and BI PP = 1). All the accessions of *Coptis* formed a monophyletic group with high support. The two samples of the new species (*C.austrogaoligongensis* C. L. Long & Z. Cheng, sp. nov.) were clustered into one clade and sister to the C.teetasubsp.teeta and C.teetasubsp.lohitensis clade (Fig. 1). Morphological PCA results show that the three species are obviously divided into three clusters, with an interpretation of 39.9% for PCA1 and 23.6% for PCA2. The long distance between the three species indicates obvious differences between them (Fig. 2).

### ﻿Taxonomic treatment

#### ﻿ Coptis austrogaoligongensis

Taxon classificationPlantaeRanunculalesRanunculaceae

C. L. Long & Z. Cheng sp. nov.

5D151864-F37C-51D9-B37B-78B4CE209A0D

urn:lsid:ipni.org:names:77345372-1

**Figure 3.**
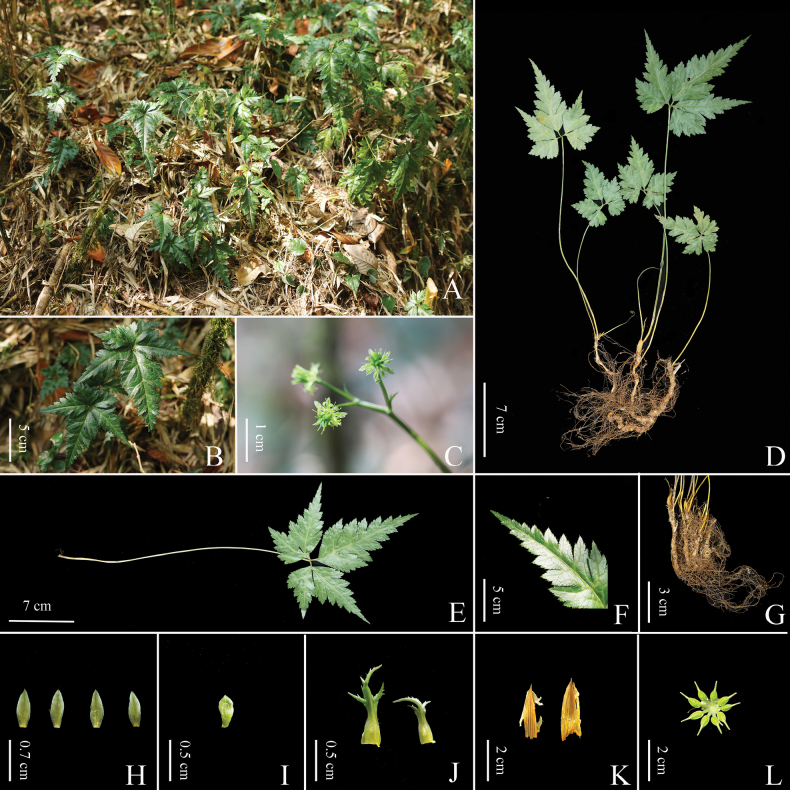
*Coptisaustrogaoligongensis* C. L. Long & Z. Cheng, sp. nov. **A** species habitat **B** leaf **C** plant in florescence stage **D** whole plant **E** petiole **F** margin with sparsely upturned spiny hairs **G** rhizome and fibrous roots **H** calyx **I** petal **J, K** bract **L** fruit. Photos by Zhuo Cheng & Jiahua Li.

**Figure 4.**
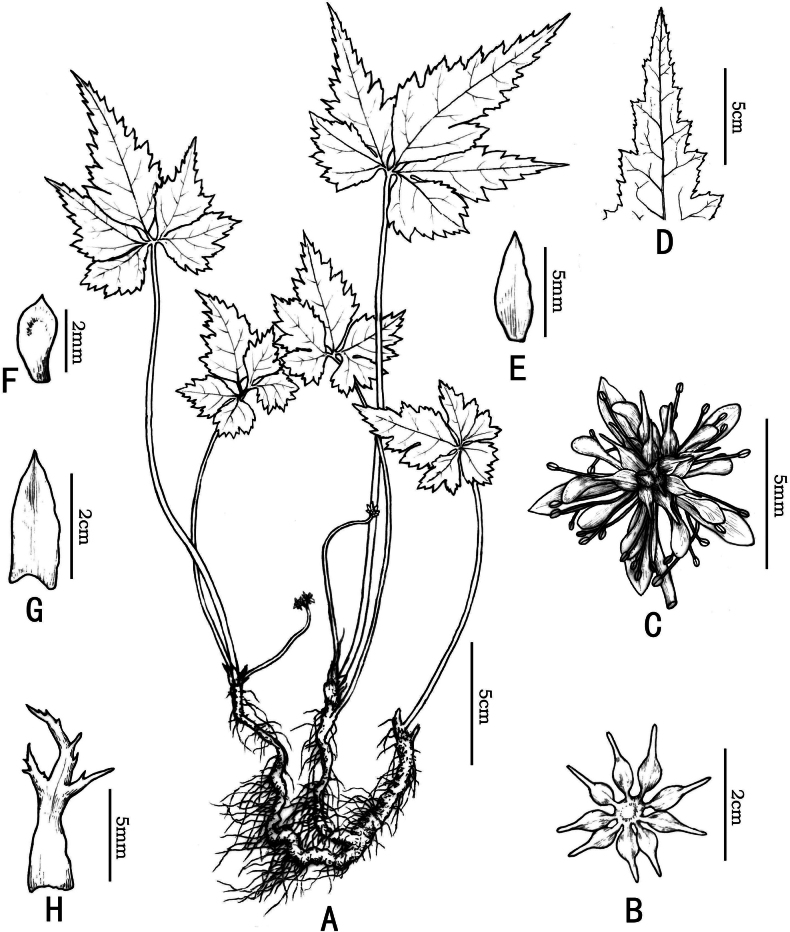
*Coptisaustrogaoligongensis* C. L. Long & Z. Cheng, sp. nov. **A** species habitat **B** Fruit **C** Flower **D** margin with sparsely upturned spiny hairs **E** calyx **F** petal **G** bract **H** bract. Drawn by Xinchen Qu.

Figs 3 , 4 “南高黎贡黄连”(Nan Gao Li Gong Huang Lian)

##### Type.

China, Yunnan Province, Dehong Dai and Jingpo Autonomous Prefecture, Yingjiang County, Zhina Township, 2444 m a.s.l., 25°15'55"N, 98°4'11"E, 20 March 2023, *Zhuo Cheng YNHL021* (holotype: KUN!; isotype: KUN!).

##### Description.

Herbs perennial, rhizomes branched, without stolons. Leaves basal, petioles 24–35 cm, glabrous. Leaf blade ovate, 7–12 × 6–12 cm, three-segmented, margin with sparsely upturned spiny hairs; central segment petiolulate (petiole 0.5–1 cm), ovate-rhombic, 6–12 × 3.5–6 cm, four-ten-lobed, lobes remote, ultimate lobes margin acute serrate, apex acute or obtuse. Scapes one to several, erect, longer or shorter than the leaves, 25–40 cm wide, glabrous, sulcate. Inflorescences terminal, often monochasial, three-five-flowered; flowers small, actinomorphic, bisexual; bracts lanceolate, palmately divided. Sepals five, greenish, long ellipsoid or lanceolate, 0.5–0.6 × 0.15–0.2 cm, sparsely puberulous. Petals spatulate, 0.15–0.25 cm long, glabrous, apex rounded to obtuse, 1/4–1/3 as long as sepals. Stamens numerous, glabrous, 2–4 mm long, outer ones slightly shorter than petals. Pistils 8–14, 3–5 mm long; follicles 4.5–9.0 mm long, stipitate; seeds ellipsoid, ca. 2–3 mm long, brown.

##### Distribution and habitat.

The only known locality of this taxon is in Zhidong Village, Zhina Township, Yingjiang County, Dehong Dai and Jingpo Autonomous Prefecture, Yunnan Province. The site is located in an open area in a primeval forest dominated by Fagaceae and Magnoliaceae. The observed population is very small, with about 100 plants growing in the bamboo forest along the roadside, accompanied by some pteridophytes. The elevation is 2400–2500 m above sea level.

##### Etymology.

The specific epithet is derived from the type locality, Gaoligongshan, Yunnan.

##### Phenology.

The species was observed flowering in February – March and fruiting in April–June.

##### Conservation status.

This species has not been recorded or described so far, and there is only one known site in Yingjiang County, which is relatively unknown to botanists. In addition, *C.austrogaoligongensis* is very small and has a short flowering period, making it easily overlooked. At the moment, without further biogeographical investigations, we can suggest that this species satisfies the IUCN 3.1 Red List CR (Critically Endangered) Criteria B1ab(ii,iii)+2ab(i,ii,iii) (IUCN 2012), which has an EOO (Extent of occurrence) < 100 km^2^ and AOO (Area of occupancy) < 10 km^2^, it may be classified as “critically endangered” (CR). The distribution site of *C.austrogaoligongensis* is next to the road, which causes a great risk of human disturbance and extreme weather, such as tourist activities, road building, grazing and landslides. Additionally, regional management in pursuit of economic development is likely to pose a threat through trampling and pollution of soil and water, probably causing negative impacts to the small and fragile habitat.

##### Taxonomic notes.

The new species belongs to Coptis section Chrysocoptis based on the following characters: leaves tri-lobed, leaflets sessile or short petiolate (Cheng et al. 1965; Wang 2001; Wang et al. 2022). There were seven species, one variant and one subspecies belonging to Coptis Section Chrysocoptis in China prior to the discovery of *C.austrogaoligongensis*. From the perspective of geographical distribution, C.teetasubsp.lohitensis and *C.quinquesecta* are both distributed in Yunnan, C.teetasubsp.lohitensis is mainly distributed in north Gaoligong Mountains and *C.quinquesecta* is mainly found in Jinping County, whereas *C.austrogaoligongensis* is mainly distributed in south Gaoligong Mountains. According to the key to the species of *Coptis* occurring in China (Wang 2001), the morphology of *C.austrogaoligongensis* is similar to C.teetasubsp.teeta and C.teetasubsp.lohitensis. However, *C.austrogaoligongensis* can be clearly distinguished by the following features: leaves with deep pinnate cleavages on whole lobes adjacent to each other, lateral segments equal to central one; plants without developed stolons; inflorescences with only 1–3 flowers; petals have short claws. A comparative summary of the characters that differentiate these three taxa is presented in Table 1.

**Table 1.** Distinguishing features of *C.austrogaoligongensis* in comparison with C.teetasubsp.lohitensis and C.teetasubsp.teeta.

Characters	*C.austrogaoligongensis*	*C.teeta* subsp. *lohitensis*	C.teetasubsp.teeta
Leaf blade	Ovate, 7–12 × 6–12 cm	Ovate-triangular, 6–12 × 5–9 cm	Ovate-triangular, 12–17.5 ×7.7–14.5cm
Leaves shape	lateral segments equal to central one, the petiole length of the middle lobe is 0.3–0.8 cm; leaves with deep pinnate cleavages on whole lobes adjacent to each other	lateral segments subsessile, shorter than central one, the petiole length of the middle lobe is 1–2.1 cm; obliquely ovate, unequally parted	lateral segments equal to central one, the petiole length of the middle lobe is 1.5–2.4 cm; obliquely ovate, unequally parted
Inflorescences	1–3 flowers	3–5 flowers	3–5 flowers
Petiole length	24–35 cm	8–19 cm	19–31 cm
Scape length	25–40 cm	15–25 cm	20–30 cm
Sepal number	5 or 6	5	5
Sepal shape	long ellipsoid or lanceolate, 0.5–0.6 × 0.15–0.2 cm, sparsely puberulous	elliptic, 0.75–0.8 × 0.25–0.3 cm, glabrous.	long ellipsoid or lanceolate, 0.5–0.6 × 0.2–0.25 cm, sparsely puberulous
Petal length	spatulate, 0.2–0.3 cm, glabrous, apex rounded to obtuse	spatulate, 0.54–0.59 cm, glabrous, apex rounded to obtuse	0.3–0.35 cm, glabrous, apex rounded to obtuse
Petal shape	petal with short claws	petals have long claws	petals have long claws
The length ratio of sepal vs petal	ca. 3 times	ca. 2 times	ca. 2 times
Are there any stolons	No	Yes	No

##### Additional *C.austrogaoligongensis* specimens examined.

China. Yunnan: Dehong Dai and Jingpo Autonomous Prefecture, Yingjiang County, Zhina Township, 2444 m a.s.l., 25°15'55"N, 98°4'11"E, 20 March 2023, Zhuo Cheng YNHL021, Zhuo Cheng YNHL022, Zhuo Cheng YNHL023, Zhuo Cheng YNHL024, Zhuo Cheng YNHL025, Zhuo Cheng YNHL026 (KUN!).

##### Specimens of C.teetasubsp.lohitensis examined.

China. Yunnan: Lushui County, 29 September 2009, L. Xie 83-0381(KUN); Lushui County, 20 November 2007, H. Li 24283 (PE); Longyang District, 23 April 2014, H.J. Dong et al. 935 (KUN); Fugong County, 15 March 2008, X.H. Jin & T. Zhang 071 (PE); Fugong County, 12 November 2007, H. Li 20256 (PE); Gongshan County, 27 September 1984, Qingzang team 9763 (PE).

### ﻿Key to the species of *Coptis* in China

There are eight species and one variant of *Coptis* distributed in China. An identification key is presented below.

**Table:** 

1	Leaves five-sectioned	**2**
–	Leaves three-sectioned	**3**
2	Rhizome robust; leaf blade 5.5–14 cm wide, central segment pinnately divided, apex Attenuate	***C.quinquesecta***
–	Rhizome slender; leaf blade 2–6 cm wide, central segment three-lobed, apex acute	***C.quinquefolia***
3	Leaf blade lanceolate to narrowly ovate; lateral segments 3–3.5× shorter than central segment; sepals linear-lanceolate	***C.omeiensis***
–	Leaf blade ovate to ovate-triangular; lateral segments slightly shorter than central segment; sepals lanceolate, elliptic, or narrowly ovate	**4**
4	Petals spatulate	**5**
–	Petals lanceolate to linear-lanceolate	**8**
5	Inflorescences three–five-flowered	**6**
–	Inflorescences more than five-flowered	***C.huanjiangensis***
6	Sparse lobes of leaf, long petals clawed	**7**
–	Close lobes of leaf; without stolons; short claws in petals	***C.austrogaoligongensis***
7	stolons developed	**C.teetasubsp.lohitensis**
–	stolons absent	**C.teetasubsp.teeta**
8	Leaf segment lobes ± contiguous to each other; stamens ca. 1/2 as long as petals	***C.deltoidea***
–	Leaf segment lobes remote; outer stamens slightly shorter than petals	**9**
9	Sepals 9–13 mm, ca. 2× as long as petals	**C.chinensisvar.chinensis**
–	Sepals ca. 6.5 mm, slightly longer than petals	**C.chinensisvar.brevisepala**

## Supplementary Material

XML Treatment for Coptis austrogaoligongensis

## ﻿Additional information

### Author ORCIDs

Zhuo Cheng https://orcid.org/0000-0001-7807-2571

Chunlin Long https://orcid.org/0000-0002-6573-6049

### Data availability

All of the data that support the findings of this study are available in the main text or Supplementary Information.

## Supplementary materials

Supplementary material 1 Supplementary information Data type xlsx Explanation note **table S1.** Accession numbers of 21 sequenced or downloaded chloroplast genomes in this study. **table S2.** List of analyzed morphological traits. Data type: table (excel file). https://binary.pensoft.net/file/1095770This dataset is made available under the Open Database License (http://opendatacommons.org/licenses/odbl/1.0/). The Open Database License (ODbL) is a license agreement intended to allow users to freely share, modify, and use this Dataset while maintaining this same freedom for others, provided that the original source and author(s) are credited.Zhuo Cheng, Jiahua Li, Congli Xu, Lixiang Zhang, Qiangbang Gong, Chunlin Long
